# Low Dose and Long Term Toxicity of Sodium Arsenite Caused Caspase Dependent Apoptosis Based on Morphology and Biochemical Character

**Published:** 2012-12-12

**Authors:** Mohammad Hussein Abnosi, Zahra Jafari Yazdi

**Affiliations:** Department of Biology, Faculty of Sciences, Arak University, Arak, Iran

**Keywords:** Apoptosis, Cell Viability, Mesenchymal Stem Cell, Rat, Sodium Arsenite

## Abstract

**Objective:**

Although arsenite is toxic it is currently recommended for the treatment of malignancies. In this study the effects of sub-micromolar concentrations of sodium arsenite on the viability, morphology and mechanism of cell death of rat bone marrow mesenchymal stem cells (BMCs) over 21 days was investigated.

**Materials and Methods:**

In this experimental study, BMCs were extracted in Dulbecco’s Modified Eagles Medium (DMEM) containing 15% of fetal bovine serum (FBS) and expanded till the 3^rd^ passage. The cells were treated with 1, 10, 25, 50, 75 and 100 nM of sodium arsenite for 21 days and the viability of the cells estimated using 3-(4, 5-dimethylthiazol-2-yl)-2, 5 diphenyl tetrazolium (MTT) and trypan blue staining. Cells were then treated with the selected dose (25 nM) of sodium arsenite to determine their colony forming ability (CFA) and population doubling number (PDN). Morphology of the cells was studied using florescent dyes, and the integrity of the DNA was investigated using the comet assay and agarose gel electrophoresis. The terminal deoxynucleotidyl transferase dUTP nick end labeling (TUNEL) and the caspase 3 assay were then applied to understand the mechanism of cell death. Data was analyzed using one way ANOVA, Tukey test.

**Results:**

A significant reduction of viability, PDN and CFA was found following treatment of BMCs with 25 nM sodium arsenite (p<0.05). Cytoplasm shrinkage and a significant decrease in the diameter of the nuclei were also seen. Comet assay and agarose gel electrophoresis revealed DNA breakage, while positive TUNEL and activated caspase 3 confirmed the apoptosis.

**Conclusion:**

A low concentration of sodium arsenite (25 nM) caused reduction of viability due to induction of apoptosis. Therefore, long term exposure to low dose of this chemical may have unwanted effects on BMCs

## Introduction

High levels of arsenic in drinking water and its associated adverse health effects are found in many developing areas around the world, including China, India, Mexico, and Bangladesh (1). Arsenic, as trivalent (AS^3+^) and pentavalent salt (AS^5+^), is released into the environment via agricultural, industrial and medical applications (2). The trivalent salt of arsenic (arsenite) is considered more toxic (3) and it has been suggested that sodium arsenite causes genetic and epigenetic changes in mouse testicular leydig cells (1). Sodium arsenite in micromolar concentrations also induces apoptosis in different types of cells such as rat midbrain neuroepithelial (4), CD4^+^ T cells (5), human neutrophils (6), Gclm mouse embryo fibroblasts (7) and human bone marrow mesenchymal stem cells (8). Sodium arsenite readily reacts with the thiol group of enzymes, receptors or coenzymes (9, 10),which may inhibit important biochemical events that could alter cellular redox status and eventually lead to cytotoxicity. Some other mechanisms including genotoxicity, alteration in DNA repair and methylation, oxidative stress, co-carcinogenesis, and tumor promotion (1, 2, 11) have also been reported in relation to arsenite toxicity.

Arsenic has been used in medicine for over 2000 years, and is still applied in diverse treatments (12). It has been suggested that interactions of arsenic with cellular proteins at submicromolar concentrations are protective, while those at higher concentrations are toxic and potentially pro-carcinogenic (13). Recently the American Food and Drug Administration (FDA) recognized the value of sodium arsenite in the treatment of malignancies in patients (14, 15) and the presence of <5µM of sodium arsenite in the serum of these patients has been reported (16, 17).

Multipotent rat bone marrow mesenchymal stem cells (MSCs) are a heterogeneous population of nonhematopoietic stem cells representing <0.01-0.001% of the total nucleated bone marrow (18). The MSCs are able to differentiate into osteoblasts, chondrocytes and adipocytes, therefore they are considered as cellular backup for bone regeneration and remodeling during homeostasis and repair (19). Yadav et al. in their report showed that a high concentration (>5µM) of sodium arsenite affects viability, DNA synthesis, morphology, the cell cycle and apoptosis of human bone marrow mesenchymal stem cells (hMSCs) (8). In our own previous work we have shown that the treatment of rat bone marrow mesenchymal stem cells for 36 and 48 hours with 0.1, 0.5 and 2.5 µM of sodium arsenite also caused morphological changes and a significant reduction in viability (20). However, there are no data available on the effect of lower doses (nM) of sodium arsenite on MSCs over long periods of exposure. The current use of sodium arsenite and its effectiveness in the treatment of acute promyelocytic leukemia (APL) (21) and acute lymphoma-type adult T-cell leukemia (ATL) (22), raises the question regarding the effects of post-therapy levels of arsenite on MSCs. Therefore, in the present study we investigate the effect of a submicromolar concentration of sodium arsenite over a period of 21 days on the viability, morphology and mechanism of cell death in rat MSCs.

## Materials and Methods

### Marrow cell culture

This experimental study was approved by the Ethics Committee of Arak University. Wistar rats (6-8 weeks old) were purchased from the Pasteur Institute, Iran and kept under standard conditions of light, temperature and food in the animal house of Arak University. Seven male rats were deeply anesthetized with ether and their femur and tibia were removed surgically. Under sterile conditions the bone marrow was flushed out using 3 ml of Dulbecco’s Modified Eagles Medium (DMEM) supplemented with 15% FBS and penicillin/streptomycin (GIBCO Company, Germany) in a 15 ml centrifuge tube. The tube was centrifuged (2500 rpm) for 5minutes at room temperature and the cell pellet was homogenized with 1ml fresh culture medium then transferred into a 25 ml culture flask. After 24 hours, unattached cells were washed off with phosphate buffer saline (PBS^+^) (containing Mg^+2^ and Ca^+2^ chloride) and adhering cells were allowed to grow for 10-14 days, with renewal of culture medium every three days. Cells were trypsinized (Trypsin/EDTA solution; Sigma, Germany) at 90% confluence and subcultured at a density of 105 cells in 25 ml plastic flasks up to the 3^rd^ passage.

### Exposure to sodium arsenite

The cells were plated in appropriate culture dishes and allowed to attach for 24 hours. Then in the presence of a control group the cells were exposed to 1, 10, 25, 50, 75 and 100 nM of sodium arsenite (Merck Company, Germany). The Number of the plated cells and time of exposure was calculated according to the nature of the test and dimensions of the culture dish. These details are provided wherever necessary in descriptions of the assays below.

### Cell viability assays

#### Trypan blue exclusion assay

MSCs were seeded at a density of 5000 per well in 6-well culture plates and after 24 hours culture media containing different concentrations of sodium arsenite were added to the respective wells. The cells were kept for 21 days with an exchange of culture media every 3 days. After the treatment period the cells were washed with PBS and harvested with Trypsin/EDTA followed by centrifugation at 2500 rpm for 5 minutes, then the cell pellet was re-suspended in 1ml of fresh culture media. 10 µl of the cell suspension was stained with an equal volume of trypan blue (Sigma company, Germany) and incubated for 2 minutes at 37℃. The total number of viable cells was estimated using a hemocytometer chamber.

#### 3-(4, 5-dimethylthiazol-2-yl)-2, 5 diphenyl tetrazolium (MTT) assay

Cell viability was also quantitatively determined by MTT assay. The MSCs were seeded in 96 well plates at a density of 500 cells per well and sodium arsenite treatment was carried out as in the previous test. After the treatment period, cells were washed with PBS and 10 µl of MTT/100 µl of FBS free culture media were added and the plate incubated for 4 hours. In viable cells the yellow tetrazolium was converted to formazan crystal by mitochondrial succinate dehydrogenase enzyme. The resulting crystals were then dissolved in the 100 µl of dimethyl sulfoxide (DMSO) (Sigma Company, Germany) and absorbance was measured at 505 nm using an ELISA reader (SCO diagnostic, Germany). After plotting the standard graph, using the linear formula Y=0.016X + 0.037 with R^2^=0.996 the numbers of viable cells were calculated, where Y stands for absorbance and X stands for number of viable cells (20).

#### Selection of LD_50_ and further investigation

Based on the results of the viability test the 25 nM concentration of sodium arsenite was selected as the LD_50_; as at this concentration approximately 50% of the cells died during the 21 days of treatment. Therefore further all investigations were carried out using a concentration of 25 nM sodium arsenite.

#### Colony forming assay

To determine the colony forming ability of the cells, 4 ×10^4^ MSCs were seeded in 3cm^2^ Petri dishes. After 24 hours during which the attachment of the cells to the dishes was ensured, the medium was removed and exchanged for culture medium containing 25 nM of sodium arsenite for 7, 14 and 21 days. Cells were then washed with PBS- and stained with crystal violet (Sigma Company, Germany) to visualize the colonies. The number and diameter of the colonies were estimated under an inverted microscope.

#### Population doubling number (PDN)

To determine PDN, 4 × 10^4^ cells were plated in 3 cm^2^ plates. After 24 hours the medium was removed and exchanged for culture medium containing 25 nM of sodium arsenite and incubated for 5, 10, 15, 21 days. After the treatment periods the cells were washed with PBS and harvested using trypsin/EDTA. The cells were then counted and PDNs were calculated using the equation PDN= (logN/N_0_ × 3.31) where N is the number of cells at the end of the culture period and the N_0_ is the number of the cells plated.

#### Morphology

The attached MSCs in a 6-well plate were treated with 25 nM of sodium arsenite for 21 days. After the treatment period the cells were washed with PBS and incubated with 5 µl of Hoechst (1mg/ml) (Sigma Company, Germany) for 5 minutes to stain the chromatin. Using Hoechst fluorescent dye, the morphology of the cell nuclei was investigated, and the diameters of the nuclei of the control and treated cells were measured in µm with the help of Motic Image software (Micro Optical Group Company version 1.2). Further to study the morphology of the cell cytoplasm, attached cells were incubated with 10 µl of acridine orange (0.5 mg/ml) for 5 minute separately. The cells after staining with Hoechst and acridine orange were observed under inverted fluorescence microscopy (Olympus, IX70, Japan).

#### Comet assay

DNA breakage in control as well as sodium arsenite (25 nM) treated cells was investigated using single-cell gel electrophoresis (comet assay) as described by Lynn et al. (23) with some modification. Briefly, after 21 days of treatment MSCs were harvested and embedded in 1% low melt agarose (Fermentas Company, Iran) gel at a density of 1×10^6^ cells/ml, and spread on a microscopic slide previously coated with normal melting agarose. The slides were immersed in ice-cold lysis buffer(10 mM Tris-HCL, 2.5M NaCl, 100 mM Na_2_ EDTA, 1% sodium N-lauroyl sarcosinate, pH=10) for 1 hour at 4℃. Then the cellular DNA was denatured in electrophoresis buffer (300 mM NaOH AND 1 mM Na_2_EDTA) for 20 minutes at room temperature and electrophoresis was carried out for 20 minutes at constant voltage (25 V). The slides were washed in distilled water and renatured in 0.4M Tris-HCl (pH=7.5). The whole procedure was carried out under indirect light and the slides were then stained with ethidium bromide (2 µg/ml) and examined under an inverted fluorescence microscope (Olympus, IX70).

#### Agarose gel electrophoresis of DNA

To detect the DNA breakage on agarose gel, total cellular DNA was extracted from arsenite treated and controls MSCs using a commercial kit (CinnaGen Company, Iran). Extracted DNA samples were electrophoresed on a 2% agarose gel in the presence of ethidium bromide at a constant voltage of 75 V for 1 hour. A molecular weight marker of 100 to 1500 base pairs (Fermentas Company, Iran) was run with the samples. Bands were visualized under UV light and a digital image of the gel was captured using an IS-1000 gel documentation system (Syngene Company, England).

#### Terminal deoxy nucleotidyl transferase-mediated dUTP nick-end labeling (TUNEL) assay

Apoptotic cells in treated and control samples were end-labeled *in situ* with the TUNEL technique using an *in situ* Cell Death Detection Kit POD (Roche, Germany, LOT: 13965100) according to the manufacturer’s instructions. In brief, cells attached to the 24 well plates were fixed with 4% paraformaldehyde in PBS (Freshly prepared) for 1 hour at 25℃. Slides were then washed with PBS and endogenous peroxidase was blocked by incubating the slide in a blocking solution containing 3% H_2_O_2_ in methanol for 10 minutes at 25℃. Slides were rinsed with PBS for 2 minutes and incubated in permeabilization solution (TritonX-100 and sodium citrate in water, freshly prepared) at 4℃ for 2 minutes. 50µl TUNEL Reaction Mixture (50 µl Enzyme solution and 450 µl label solution, freshly prepared) was added to the samples then covered with a layer of parafilm and incubated for 60 minutes at 37℃ under humid conditions in the absence of light. A negative control was prepared by incubating the sample with only 50 µl of label solution without the enzyme terminal transferase. After washing the wells three times with PBS, the plate was incubated with 50 µl of anti-fluorescent antibody conjugated with horseradish peroxidase (POD) for 30 minutes at 37℃ in a humidified chamber and finally treated with diaminobenzidine (DAB) in the presence of H_2_O_2_ for 30 minutes in the dark. After washing the slides with PBS, they were observed by light microscope under magnification (×20).

#### Immunochemical staining of activated caspase 3

Detection of cleaved caspase 3, a key executioner of apoptosis, was performed using a SignalStain IHC detection kit (Chemicon, Germany, LOT# 0605029945). Cells were seeded onto a 12-well plate and after 21days of treatment they were fixed with 4% paraformaldehyde and immunochemical staining was carried out according to the manufacturer’s instructions. Briefly, to prevent nonspecific binding, the cells were immersed in a blocking solution for 1 hour at 25℃. Cells were then incubated with a pre-diluted primary antibody at 4℃ overnight and rinsed for 15 minutes with PBS. The cells were incubated with biotinylated secondary antibody for 30 minutes at room temperature, and rinsed for a further 15 minutes with PBS. The cells were then stained and counterstained according to the manufacturer’s protocol with DAB and hematoxylin respectively. The cells were observed under a light microscope equipped with a digital camera with magnification (×20). Cells exhibiting the brown cytoplasmic stain were considered positive for activated caspase-3.

#### Statistical analysis

Statistical evaluation of the data was performed using one-way analysis of variance (ANOVA), Tukey test with the help of SPSS. Results were shown as mean ± SD and p<0.05 was accepted as the minimum level of significance.

## Results

### Cell viability

Treatment of the cells with sodium arsenite for 21 days showed a significant reduction in viability (p<0.05) from 10 nM upwards (Table 1). Results

### Colony forming assay

**Fig 1:**
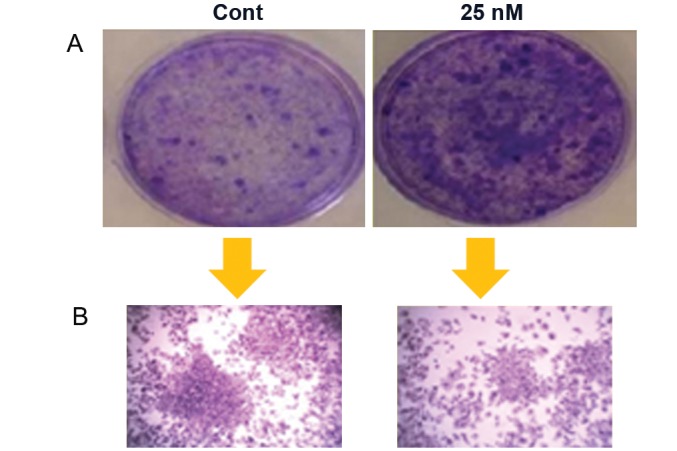
A. Photograph of the culture plates showing the visual difference between the numbers of colonies in control and treated wells. B. Microscopic picture showing the visual differences between the colony size in control and treated wells.

Treatment of the cells with 25 nM of sodium arsenite caused a highly significant reduction (p<0.05) in the number and diameter of the colonies at days 7, 14 and 21 (Tables 2, 3). Based on the reduction in color intensity, figure 1 also showed the reduction in number and diameter of the colonies at day 21 compared to the control group of cells.

**Table 1:** Viability of the mesenchymal stem cells after 21 days of treatment with 0, 1, 10, 25, 50, 75 and 100 nM of sodium arsenite based on Trypan blue staining and MTT assay


Doses (nM) of sodium arsenite	Trypan blue (Percentage of viable cells)	MTT assay (Number of viable cells)

0	096.6^a^ ± 1.1	27620^a^ ± 2199.8
1	94.4^a^ ± 1.3	26840^a^ ± 1901.7
10	84.2^b^ ± 1.3	21410^b^ ± 1104.9
25	59.1^c^ ± 2.7	14971^c^ ± 833.1
50	21.2^d^ ± 2.9	6360^d^ ± 681.8
75	13.2^e^ ± 0.7	5960^d^ ± 407.5
100	11.2^e^ ± 0.1	5906^d^ ± 342.5

Values are means ± SD. Means with the same letter code in each column do not differ significantly from each other (ANOVA, Tukey test, p>0.05).

**Table 2:** Mean number of colonies after 7, 14 and 21 days of treatment with 25 nM of sodium arsenite in comparison with the control


Days	7	14	21
Dose(nM)			

0	122.2^a^ ± 3/1	180.2^a^ ± 2.5	457.4^a^ ± 3.4
25	41.0^b^ ± 1.6	71.0^b^ ± 1.4	84.4^b^ ±2.7


Values are means ± SD. Mean values with the different letter code in each column differ significantly from each other (ANOVA, Tukey test, p<0.05).

**Table 3:** Mean diameter (mm) of the colonies after 7, 14 and 21 days of treatment with 25 nM of sodium arsenite in comparison with the control


Days	7	14	21
Dose(nM)			

0	0.29^a^ ± 0.1	0.44^a^ ± 0.1	0.58^a^ ± 0.1
25	0.17^b^ ± 0.1	0.21^b^ ± 0.1	0.29^b^ ± 0.1


Values are means ± SD. Mean values with the different letter code in each column differ significantly from each other (ANOVA, Tukey test, p<0.05).

### Population doubling number

Maximum population doubling of the control group of cells occurred on day 5 and day 10, whereas on day AB15 and 20 no considerable rise in the doubling number could be observed. Sodium arsenite caused a significant reduction (p<0.05) in the MSCs population doubling number on days 5, 10, 15 and 21 in comparison to the control group of cells (Table 4).

**Table 4:** Mean population doubling number of MSCs after 5, 10, 15 and 21 days of treatment with 25 nM of sodium arsenite in comparison with the control


Days	5	10	15	21
Dose(nM)				

0	2.88^a^ ± 0.03	4.17^a^ ± 0.02	4.55^a^ ± 0.15	4.81^a^ ± 0.13
25nM	1.84^b^ ± 0.05	2.18^b^ ± 0.07	2.35^b^ ± 0.07	2.63^b^ ± 0.04


Values are means ± SD.Mean values with the different letter code in each column differ significantly from each other (ANOVA, Tukey test, p<0.05).

### Morphology

**Fig 2:**
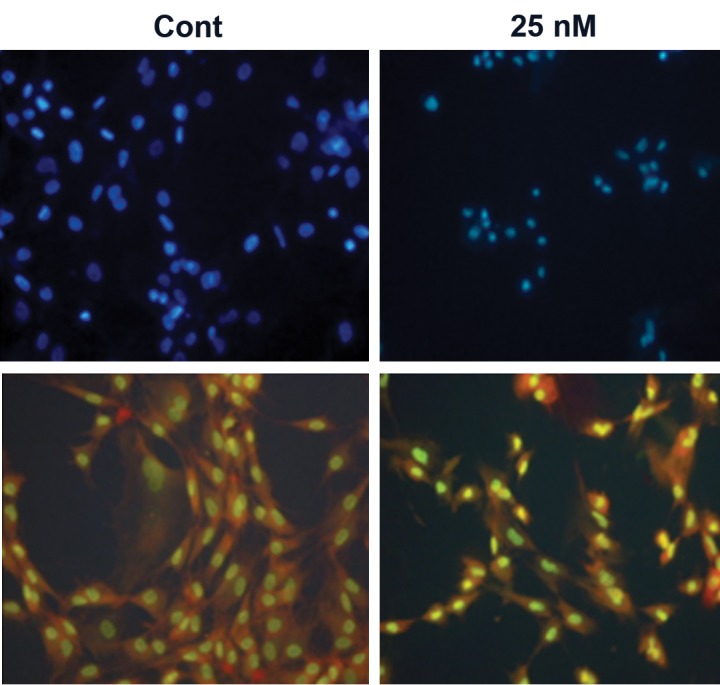
Fluorescent staining, A. cells stained with Hoechst. Sodium arsenite treatment caused nuclear size reduction and chromatin breakage compared with the control. B. cells stained with acridine orange. Control cells showed the typical morphology of mesenchymal stem cell while sodium arsenite caused roundness of the cells and cytoplasm shrinkage (×40 magnification).

Morphological study of the nuclei of mesenchymal stem cells treated with 25 nM of sodium arsenite after 21 days showed chromatin condensation and nuclear breakage (Fig 2A). Also a highly significant reduction (p<0.001) in mean diameter of the nuclei of the treated cells (6.47 ± 0.30) was observed compared with control cells (11.01 ± 0.39). It can be also noticed that sodium arsenite at this concentration caused remarkable changes in the morphology of the cytoplasm (Fig 2B) such as cell roundness and cytoplasm shrinkage and in some cells complete disappearance of the cytoplasm content compared with the control group of cells.

### Comet assay

**Fig 3:**
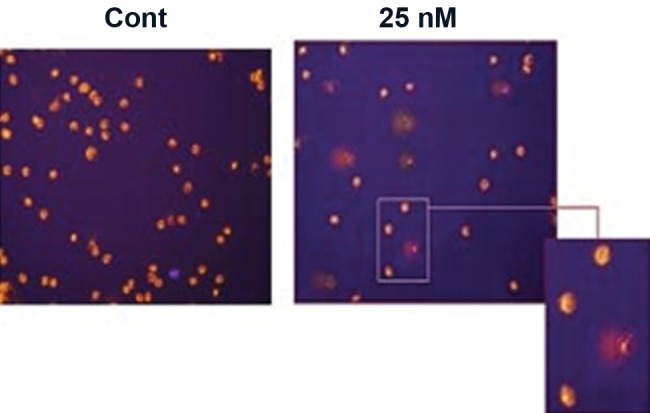
The single cell gel electrophoresis (comet assay) of the control and treated cells (25 nm) after 21 days. No DNA break was observed in control (cont), The DNA is fragmented and a tail can be observed due to DNA breakage in the treated cells (25 nM), (×20).

A very sensitive method called "Single-cell alkaline gel electrophoresis" or "Comet assay" was adopted to investigate the state of the DNA. This method showed that the treatment of MSCs with 25nM of sodium arsenite for 21 days caused the DNA of the cell to break into large pieces in which a tail is formed behind the cells to indicate the comet formation (Fig 3).

### Agarose gel electrophoresis of DNA

**Fig 4:**
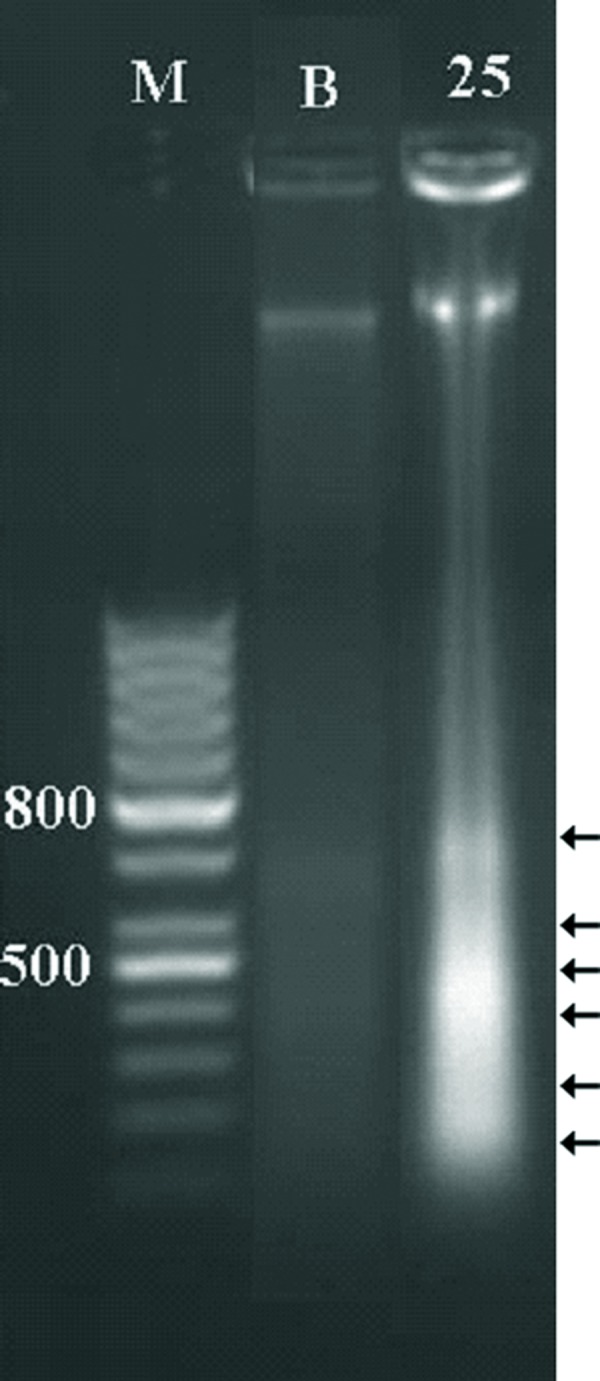
DNA gel electrophoresis of the control and treated cells after 21 days. M. marker, B. control cells, which the total DNA is intact and 25. cells treated with 25nM of sodium arsenite showing nucleosome breakage and ladder formation (arrows).

Agarose gel electrophoresis of the total DNA extracted from treated cells with 25 nM of sodium arsenite showed breakage of the DNA in the shape of a ladder. The ladder starts from the smallest piece of DNA with an approximate size of 100 bp to the largest, which may be considered as a single nucleosome up to a length of DNA that contains many nucleosomes (Fig 4).

### TUNEL assay

**Fig 5:**
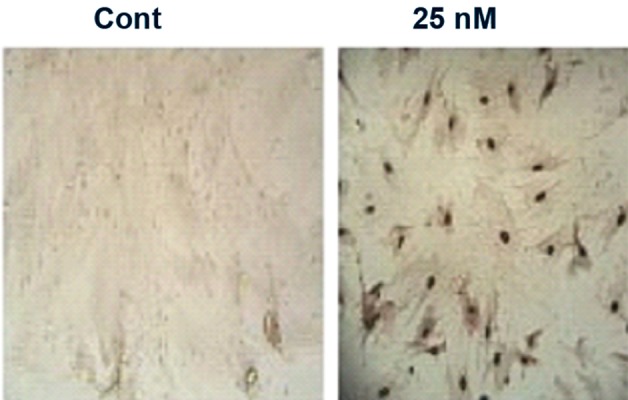
TUNEL assay of the control and treated cells after 21 days. No DNA breakage can be seen in the control cells (Cont). In the treated cells the DNA is broken, thus the nuclei of the cells appear brown (25 nM), (×20 magnifications).

In accordance with the results of the chromatin staining, comet assay and DNA gel electrophoresis, which showed chromatin condensation and breakage of DNA, the TUNEL assay also showed a clear increase in the number of TUNEL-positive cells in the treated wells. According to the manufacturer wherever a breakage appears in the chromatin, nucleotidyl transferase builds up a poly dUTP which forms the base for attachment of the POD conjugated antibody. Thus, conjugated POD oxidized the DAB and a brown colored deposition was observed in the sodium arsenite treated cells compared to the control group of cells (Fig 5).

### Immunochemical staining of activated caspase 3

**Fig 6:**
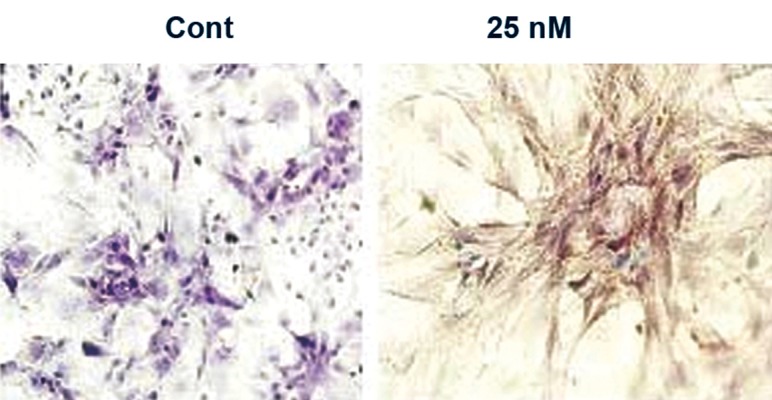
immunochemical staining of caspase 3. No activated caspase 3 in the control group of cells was observed (Cont), activated caspase 3 is seen in the cells treated with 25 nM of sodium arsenite, thus the cytoplasm of the cells appear brown due to color of oxide DAB (25 nM), (×20 magnification).

Immunocytochemistry of caspases showed that the MSCs treated with 25 nM of sodium arsenite for 21 days have activated caspase 3 in their cytoplasm. In figure 6 a deep brown color due to deposition of oxidized 3,3'-diaminobenzidine (DAB) was observed in the cytoplasm of treated cells compared to control cells. According to the manufacturer, horseradish peroxidase conjugated to secondary antibody attaches to activated caspase 3 and converts reduced DAB to oxidized DAB in the presence of H_2_O_2_. Caspase 3, an executioner caspase, which is activated by caspase 9 and 8 via the intrinsic and extrinsic pathways respectively, thus brings about the activation of nucleases and finally breaks the DNA in the cell.

## Discussion

Previous studies have shown that micromolar concentrations of sodium arsenite after 12 to 48 hours cause significant reductions in the viability of human and rat BMSCs in a time and dose dependent manner (8, 20). A sodium arsenite cytotoxicity study carried out by Sidhu and et al on embryonic primary rat midbrain neuroepithelial cells at 24 to 48 hours also confirmed a concentration- and time-dependent reduction of viability (4). In line with previous work but for the first time, the present study showed that even sub micromolar concentrations of sodium arsenite over a longer period of time can cause a dose dependent reduction of viability. Sodium arsenite in micromolar concentrations has been reported to be effective in APL and ATL therapy (21, 22) and the American Food and Drug Administration has recommended the use of this chemical in malignancy therapy (14, 15). However it seems that after sodium arsenite treatment the remaining low concentration of this chemical in the blood and other tissues over a longer course of time can affect the wellbeing of the MSCs. Although, researchers have shown the effectiveness of sodium arsenite as a therapeutic agent, a question remains as to whether it should be considered as such given that it can put the health of the adult stem at risk. Studies have shown that 1, 2.5, and 5 µM of sodium arsenite up to 48 hours causes no detectable toxicity to H9C2 myoblast cells and hMSCs (8, 24) whereas our study revealed that even nanomolar concentrations of sodium arsenite over a long time can cause a significant reduction in rat bone marrow mesenchymal stem cell viability, based on Trypan blue and MTT as well as PDN and colony forming unit (CFU) tests. It should therefore be concluded that time is a very important factor to be considered in the cytotoxicity effect of sodium arsenite.

Morphological study of the MSCs, using fluorescent dye showed that 25 nM of sodium arsenite after 21 days caused the condensation and breakage of cell nuclei as well as cytoplasm shrinkage. Arsenite exposure, in µM concentrations over a short period of time (6 hours) has been reported to cause changes in morphology and cell growth retardation in Chinese hamster V79 fibroblast cells (25). In addition, it has been reported that sodium arsenite induced morphological apoptosis in both HeLa and MCF-7 tumor cells as well as hMSCs in a dose-dependent manner (8, 26). Although the exact mechanism of sodium arsenite toxicity is not well known, the morphological changes observed in the cells may be due to the elevated level of free radicals (23), reduction in activity of the DNA repairing enzymes (27) and induction of apoptosis as well as cell cycle inhibition (4). If the long term toxicity of a low dose of sodium arsenite can induce morphological changes in the nuclei and cytoplasm of the mesenchymal stem cells then it might be a risk factor which affects bone health in industrial or other contaminated areas.

Previously it was reported that a high concentration (>5µM) of sodium arsenite over 24 to 48 hours causes apoptosis in hMSCs by altering Bcl-2 family proteins (8). Our study showed that a concentration of 25nM of sodium arsenite after 21 days caused caspase dependent apoptosis based on observations of the nuclear morphology and biochemical character of the cells, such as DNA damage confirmed by comet assay and agarose gel electrophoresis, and TUNEL assay, in addition to activated caspase 3 in the cell cytoplasm. Apoptosis, or programmed cell death, is a distinct mechanism where the cell undergoes shrinkage and nuclear breakage with formation of nucleosomal fragments due to chromatin cleavage (28). In spite of other reports which show sodium arsenite is able to induce apoptosis at high concentrations, the results of this study reveal that even a low dose of this chemical over the course of time is able to induce apoptosis in rat mesenchymal stem cells.

## Conclusion

The results of this study showed that even a low dose of sodium arsenite (25 nM) over a longer course of time can cause a significant reduction in the viability of MSCs, and the mechanism of cell death is caspase dependent apoptosis. Therefore even if sodium arsenite has been recommended for therapy, we feel that more research needs to be to be carried out to understand the extent of the molecular damage it inflicts on other adult stem cells over a longer period of time at doses lower than those used in therapy.
